# Supplementary material for: Pan-Canadian survey on the impact of the COVID-19 pandemic on cervical cancer screening and management: cross-sectional survey of healthcare professionals
Source: eLife. 2023 Jun 28;12:e83764. doi: 10.7554/eLife.83764 (PMC10368419; doi:10.7554/eLife.83764)
Supplement: Supplementary file 1. — Hard copy of the online survey instrument. [file elife-83764-supp1.docx]

# Pan-Canadian Survey on the Impact of the COVID-19 Pandemic on Cervical Cancer Screening and Management

The COVID-19 pandemic has caused unprecedented disruptions to cancer care by delaying diagnoses and treatment, as well as pausing clinical cancer research. We are a consortium of healthcare providers, researchers, and cancer control experts collaborating with the Division of Cancer Epidemiology at McGill University. Our goal is to assess the pandemic’s impact on cervical cancer screening in Canada, at provincial and local levels, so we can formulate solutions that would mitigate the consequences of this unprecedented disruption in one of the most key components of cancer prevention.

As a healthcare professional involved with one or more aspects of cervical cancer screening, diagnosis, management, and treatment, we need to hear from you about the positive and negative experiences you have had in your practice.

The survey, which is part of a research study led by Dr. Eduardo Franco, is divided into sections, some of which may not apply to you and your practice. Please note that you are able to go back to previous themes and revise your answers through the "previous" at the end of each page. The "Question Index" button on the top right of each page allows you to navigate between different themes of the survey. A "Not applicable to my practice" option has been added to every question; please choose that answer if a question relates to procedures with which you are not involved within your practice. It should take you up to 20 minutes to complete. Your responses are completely anonymous and will be used in aggregate, statistical form.

As a token of appreciation, a link will appear following your completion of the survey, allowing you to participate in a prize draw.

Survey Questions

Please note that questions 6 to 47 relate to cervical cancer screening and management during the COVID-19 period, with several formulated relative to the pre-COVID-19 era. For the purpose of estimating cumulative effects during the COVID-19 period of interest, please assume it to span from mid-March to mid- August 2020 (this reminder will appear at the beginning of every theme).

Informed E-consent

1. I agree to participate in the survey. I understand the purpose and nature of this survey and I am participating voluntarily.
   - Yes
   - No

Practice and Institution First, please fill in the following information relating to your practice/institution:

1. In which province/territory do you currently work?
   - Alberta
   - British Columbia
   - Manitoba
   - New Brunswick
   - Newfoundland and Labrador
   - Northwest Territories
   - Nova Scotia
   - Nunavut
   - Ontario
   - Prince Edward Island
   - Quebec
   - Saskatchewan
   - Yukon
   - Prefer not to say
2. Enter the first three letters/digits of your workplace postal code:
3. What is your professional role/responsibility? [*Select all that apply*]
   - Colposcopist
   - Colposcopy RN/RPN
   - Cytopathologist
   - General Practitioner/Family Physician
   - Gynecologist
   - Gynecological Oncologist
   - GYN Nurse
   - Nurse practitioner
   - Pathologist
   - Physician Assistant
   - Other, *please specify*:
4. Where do you practice? [*Select all that apply*]
   - University-affiliated hospital
   - Community hospital
   - Public clinic
   - Private clinic
   - Community health center
   - Other, *please specify*:

Screening Practice

# Appointment Scheduling

For the purpose of estimating cumulative effects during the COVID-19 period of interest, please assume it to span from mid-March to mid-August 2020.

1. Did you encounter any cancellations *(i.e., cancelled and not re-booked)* of

screening appointments scheduled in your practice/institution?

- - Yes
  - No
  - Don’t know
  - Not applicable to my practice

1. What percentage of all cancelled screening appointments were:

|  | 0% | 1-24% | 25-49% | 50-74% | ≥75% | Don’t know |
| --- | --- | --- | --- | --- | --- | --- |
| Cancelled by physicians or providers’  institutions | ☐ | ☐ | ☐ | ☐ | ☐ | ☐ |
| Cancelled by  patients | ☐ | ☐ | ☐ | ☐ | ☐ | ☐ |
| Converted to  telemedicine | ☐ | ☐ | ☐ | ☐ | ☐ | ☐ |

- Please make sure your answer does not exceed 100%.

1. Did you encounter any postponements *(i.e., postponed and/or re-booked)* of

screening appointments scheduled in your practice/institution?

- - Yes
  - No
  - Don’t know
  - Not applicable to my practice

1. What percentage of all postponed screening appointments were:

|  | 0% | 1-24% | 25-49% | 50-74% | ≥75% | Don’t know |
| --- | --- | --- | --- | --- | --- | --- |
| Postponed by physicians or providers’  institutions | ☐ | ☐ | ☐ | ☐ | ☐ | ☐ |
| Postponed by  patients | ☐ | ☐ | ☐ | ☐ | ☐ | ☐ |
| Converted to  telemedicine | ☐ | ☐ | ☐ | ☐ | ☐ | ☐ |

- Please make sure your answer does not exceed 100%

1. How long was the deferral period for postponed screening appointments?
   - 1 week to less than 2 weeks
   - 2 weeks to less than 4 weeks
   - 1 month to less than 2 months
   - 2 months to less than 4 months
   - 4 months to less than 6 months
   - Greater than 6 months
   - Don’t know
2. During the peak period of the pandemic, did your practice/institution allow in-person consultation appointments?
   - Yes
   - No
   - Don’t know
   - Not applicable to my practice
3. During the pandemic period, did you feel you were informed about the latest evidence-based recommendations and/or guidelines related to cervical

cancer screening (*i.e., from sources you would have relied on during the pre- COVID-19 era*)?

- - Yes
  - No
  - Don’t know
  - Not applicable to my practice

1. How often have you received updates in recommendations and/or guidelines from your institution/provincial body that modify scheduling/performing cervical cancer screening procedures?
   - Every week
   - Every 2 weeks
   - Every month
   - Every 2 months
   - > 2 months
   - Never
   - Don’t know
   - Not applicable to my practice

# Screening Tests

For the purpose of estimating cumulative effects during the COVID-19 period of interest, please assume it to span from mid-March to mid-August 2020.

1. Irrespective of the pandemic situation, which test(s) does your practice/institution use for primary cervical cancer screening?

|  | Yes | No | Don’t know | Not applicable to my practice |
| --- | --- | --- | --- | --- |
| Pap test (conventional/LBC) | ☐ | ☐ | ☐ | ☐ |
| HPV test (Primary screening/ASCUS triage) | ☐ | ☐ | ☐ | ☐ |
| HPV/Pap co-test | ☐ | ☐ | ☐ | ☐ |

|  | Decrease by 75% or more | Decrease by 26-  74% | Decrease by 25% or less | Unaffected | Increase by 25% or less | Increase by 26-  74% | Increase by 75% or more | Don’t know | Not applicable to my  practice |
| --- | --- | --- | --- | --- | --- | --- | --- | --- | --- |
| Pap test (conventional  /LBC) | ☐ | ☐ | ☐ | ☐ | ☐ | ☐ | ☐ | ☐ | ☐ |

| HPV test (Primary screening/ASCUS  triage) | ☐ | ☐ | ☐ | ☐ | ☐ | ☐ | ☐ | ☐ | ☐ |
| --- | --- | --- | --- | --- | --- | --- | --- | --- | --- |
| HPV/Pap co-test | ☐ | ☐ | ☐ | ☐ | ☐ | ☐ | ☐ | ☐ | ☐ |

1. Were there any changes in the number of these tests carried out in your practice/institution, in comparison to the pre-COVID-19 era?
2. Were there any delays in the scheduling of these tests?

|  | Yes | No | Don’t know | Not applicable to my practice |
| --- | --- | --- | --- | --- |
| Pap test (conventional/LBC) | ☐ | ☐ | ☐ | ☐ |
| HPV test (Primary screening/ASCUS triage) | ☐ | ☐ | ☐ | ☐ |
| HPV/Pap co-test | ☐ | ☐ | ☐ | ☐ |

1. What percentage of all scheduled tests have been cancelled?

|  | 0% | 1-24% | 25-49% | 50-74% | ≥75% | Don’t  know | Not applicable  to my practice |
| --- | --- | --- | --- | --- | --- | --- | --- |
| Pap test (conventional/LBC) | ☐ | ☐ | ☐ | ☐ | ☐ | ☐ | ☐ |
| HPV test (Primary  screening/ASCUS triage) | ☐ | ☐ | ☐ | ☐ | ☐ | ☐ | ☐ |
| HPV/Pap co-test | ☐ | ☐ | ☐ | ☐ | ☐ | ☐ | ☐ |

1. What percentage of all scheduled tests have been postponed?

|  | 0% | 1-24% | 25-49% | 50-74% | ≥75% | Don’t  know | Not applicable  to my practice |
| --- | --- | --- | --- | --- | --- | --- | --- |
| Pap test (conventional/LBC) | ☐ | ☐ | ☐ | ☐ | ☐ | ☐ | ☐ |
| HPV test (Primary  screening/ASCUS triage) | ☐ | ☐ | ☐ | ☐ | ☐ | ☐ | ☐ |
| HPV/Pap co-test | ☐ | ☐ | ☐ | ☐ | ☐ | ☐ | ☐ |

1. On average, how long was the deferral period for postponed *(i.e., postponed and/or re-booked*) screening appointments?

|  | 1 week to less than 2  weeks | 2 weeks to less than 4  weeks | 1 month to less than 2  months | 2 months to less than 4  months | 4 months to less than 6  months | Greater than 6 months | Don’t know | Not applicable to my  practice |
| --- | --- | --- | --- | --- | --- | --- | --- | --- |
| Pap test  (conventional/LBC) | ☐ | ☐ | ☐ | ☐ | ☐ | ☐ | ☐ | ☐ |
| HPV test (Primary screening/ASCUS  triage) | ☐ | ☐ | ☐ | ☐ | ☐ | ☐ | ☐ | ☐ |
| HPV/Pap co-test | ☐ | ☐ | ☐ | ☐ | ☐ | ☐ | ☐ | ☐ |

1. Was there any delay, due to pandemic-related factors, in forwarding test samples to the lab for processing?

|  | Yes | No | Don’t know | Not applicable to my practice |
| --- | --- | --- | --- | --- |
| Pap test (conventional/LBC) | ☐ | ☐ | ☐ | ☐ |
| HPV test (Primary screening/ASCUS triage) | ☐ | ☐ | ☐ | ☐ |
| HPV/Pap co-test | ☐ | ☐ | ☐ | ☐ |

1. On average, how long was the delay period associated with forwarding test samples to the lab?

|  | 1 week to less than 2  weeks | 2 weeks to less than 4  weeks | 1 month to less than 2  months | 2 months to less than 4  months | 4 months to less than 6  months | Greater than 6 months | Don’t know | Not applicable to my  practice |
| --- | --- | --- | --- | --- | --- | --- | --- | --- |
| Pap test  (conventional/LBC) | ☐ | ☐ | ☐ | ☐ | ☐ | ☐ | ☐ | ☐ |
| HPV test (Primary screening/ASCUS  triage) | ☐ | ☐ | ☐ | ☐ | ☐ | ☐ | ☐ | ☐ |
| HPV/Pap co-test | ☐ | ☐ | ☐ | ☐ | ☐ | ☐ | ☐ | ☐ |

# HPV Self-Sampling

HPV self-sampling refers to testing for HPV using self-collected vaginal samples at home or a clinic.

For the purpose of estimating cumulative effects during the COVID-19 period of interest, please assume it to span from mid-March to mid-August 2020.

1. Do you think that the COVID-19 pandemic will encourage/facilitate/accelerate the implementation of HPV self-sampling in cervical cancer screening programs, at a provincial and/or national level?
   - Yes
   - No
   - Maybe

- Briefly justify your answer in the comment box above

1. Are you in favor of the implementation of HPV self-sampling as an alternative screening method in your clinical practice?
   - Yes
   - No
   - Maybe

- Briefly justify your answer in the comment box above

# Colposcopy

For the purpose of estimating cumulative effects during the COVID-19 period of interest, please assume it to span from mid-March to mid-August 2020.

1. Were there any changes in the number of colposcopy-biopsy procedures carried out in your practice/institution?
   - Decrease by 75% or more
   - Decrease by 26-74%
   - Decrease by 25% or less
   - Unaffected
   - Increase by 25% or less
   - Increase by 26-74%
   - Increase by 75% or more
   - Don’t know
   - Not applicable to my practice
2. Did you encounter any cancellations *(i.e., cancelled and not re-booked)* of

colposcopy appointments scheduled in your practice/institution?

- - Yes
  - No
  - Don’t know
  - Not applicable to my practice

1. What percentage of all cancelled colposcopy appointments were:

|  | 0% | 1-24% | 25-49% | 50-74% | ≥75% | Don’t know |
| --- | --- | --- | --- | --- | --- | --- |
| Cancelled by physicians or providers’  institutions | ☐ | ☐ | ☐ | ☐ | ☐ | ☐ |
| Cancelled by  patients | ☐ | ☐ | ☐ | ☐ | ☐ | ☐ |

- Please make sure your answer does not exceed 100%

1. Did you encounter any postponements *(i.e., postponed and/or re-booked)* of

colposcopy appointments scheduled in your practice/institution?

- - Yes
  - No
  - Don’t know
  - Not applicable to my practice

1. What percentage of all postponed colposcopy appointments were:

|  | 0% | 1-24% | 25-49% | 50-74% | ≥75% | Don’t know |
| --- | --- | --- | --- | --- | --- | --- |
| Postponed by physicians or providers’  institutions | ☐ | ☐ | ☐ | ☐ | ☐ | ☐ |
| Postponed by  patients | ☐ | ☐ | ☐ | ☐ | ☐ | ☐ |

- Please make sure your answer does not exceed 100%

1. How long was the deferral period for postponed colposcopy appointments?
   - 1 week to less than 2 weeks
   - 2 weeks to less than 4 weeks
   - 1 month to less than 2 months
   - 2 months to less than 4 months
   - 4 months to less than 6 months
   - Greater than 6 months
   - Don’t know

# Screening Follow-up

For the purpose of estimating cumulative effects during the COVID-19 period of interest, please assume it to span from mid-March to mid-August 2020.

1. Was there any delay in follow-up appointments with patients regarding any of the following results?

|  | Yes | No | Don’t know | Not applicable to my practice |
| --- | --- | --- | --- | --- |
| Abnormal cytology results | ☐ | ☐ | ☐ | ☐ |
| High-grade lesions | ☐ | ☐ | ☐ | ☐ |
| Positive HPV test results | ☐ | ☐ | ☐ | ☐ |
| Positive HPV self-sampling test  results | ☐ | ☐ | ☐ | ☐ |

1. Did you encounter any cancellations *(i.e., cancelled and not re-booked)* of

follow-up appointments scheduled in your practice/institution?

- - Yes
  - No
  - Don’t know
  - Not applicable to my practice

1. What percentage of all cancelled follow-up appointments were:

|  | 0% | 1-24% | 25-49% | 50-74% | ≥75% | Don’t know |
| --- | --- | --- | --- | --- | --- | --- |
| Cancelled by physicians or providers’  institutions | ☐ | ☐ | ☐ | ☐ | ☐ | ☐ |
| Cancelled by  patients | ☐ | ☐ | ☐ | ☐ | ☐ | ☐ |
| Converted to  telemedicine | ☐ | ☐ | ☐ | ☐ | ☐ | ☐ |

- Please make sure your answer does not exceed 100%

1. Did you encounter any postponements *(i.e., postponed and/or re-booked)* of

follow-up appointments scheduled in your practice/institution?

- - Yes
  - No
  - Don’t know
  - Not applicable to my practice

1. What percentage of all postponed follow-up appointments were:

|  | 0% | 1-24% | 25-49% | 50-74% | ≥75% | Don’t know |
| --- | --- | --- | --- | --- | --- | --- |
| Postponed by physicians or providers’  institutions | ☐ | ☐ | ☐ | ☐ | ☐ | ☐ |
| Postponed by  patients | ☐ | ☐ | ☐ | ☐ | ☐ | ☐ |
| Converted to  telemedicine | ☐ | ☐ | ☐ | ☐ | ☐ | ☐ |

- Please make sure your answer does not exceed 100%

1. How long was the deferral period for postponed follow-up appointments?
   - 1 week to less than 2 weeks
   - 2 weeks to less than 4 weeks
   - 1 month to less than 2 months
   - 2 months to less than 4 months
   - 4 months to less than 6 months
   - Greater than 6 months
   - Don’t know
2. Was there any delay, due to pandemic-related factors, in receiving test results from the lab prior to follow-up with patients?

|  | Yes | No | Don’t know | Not applicable to my practice |
| --- | --- | --- | --- | --- |
| Pap test (conventional/LBC) | ☐ | ☐ | ☐ | ☐ |
| HPV test (Primary screening/ASCUS triage) | ☐ | ☐ | ☐ | ☐ |
| HPV/Pap co-test | ☐ | ☐ | ☐ | ☐ |

1. On average, how long was the delay period for receiving test results from the lab?

|  | 1 week to less than 2 weeks | 2 weeks to less than 4 weeks | 1 month to less than 2 months | 2 months to less than 4 months | 4 months to less than 6 months | Greater than 6 months | Don’t know | Not applicable to my practice |
| --- | --- | --- | --- | --- | --- | --- | --- | --- |
| Pap test (conventional/LBC) | ☐ | ☐ | ☐ | ☐ | ☐ | ☐ | ☐ | ☐ |
| HPV test (Primary screening/ASCUS triage) | ☐ | ☐ | ☐ | ☐ | ☐ | ☐ | ☐ | ☐ |
| HPV/Pap co-test | ☐ | ☐ | ☐ | ☐ | ☐ | ☐ | ☐ | ☐ |

Treatment of pre-cancerous lesions and cancer For the purpose of estimating cumulative effects during the COVID-19 period of interest, please assume it to span from mid-March to mid-August 2020.

1. Did you observe any changes in the number of procedures completed in your practice/institution?

|  | Decrease by 75% or more | Decrease by 26-  74% | Decrease by 25% or less | Unaffected | Increase by 25% or less | Increase by 26-  74% | Increase by 75% or more | Don’t know | Not applicable to my  practice |
| --- | --- | --- | --- | --- | --- | --- | --- | --- | --- |
| Cold knife  conization | ☐ | ☐ | ☐ | ☐ | ☐ | ☐ | ☐ | ☐ | ☐ |
| Other  excisional (e.g., LEEP) | ☐ | ☐ | ☐ | ☐ | ☐ | ☐ | ☐ | ☐ | ☐ |
| Ablative  procedures | ☐ | ☐ | ☐ | ☐ | ☐ | ☐ | ☐ | ☐ | ☐ |
| Hysterectomy | ☐ | ☐ | ☐ | ☐ | ☐ | ☐ | ☐ | ☐ | ☐ |
| Chemotherapy | ☐ | ☐ | ☐ | ☐ | ☐ | ☐ | ☐ | ☐ | ☐ |
| Radiation | ☐ | ☐ | ☐ | ☐ | ☐ | ☐ | ☐ | ☐ | ☐ |

1. Did you encounter any cancellations/postponements of treatment

procedures scheduled in your practice/institution?

|  | Yes | No | Don’t know | Not applicable to my practice |
| --- | --- | --- | --- | --- |
| Cold knife conization | ☐ | ☐ | ☐ | ☐ |
| Other excisional (e.g., LEEP) | ☐ | ☐ | ☐ | ☐ |
| Ablative procedures | ☐ | ☐ | ☐ | ☐ |
| Hysterectomy | ☐ | ☐ | ☐ | ☐ |
| Chemotherapy | ☐ | ☐ | ☐ | ☐ |
| Radiation | ☐ | ☐ | ☐ | ☐ |

1. What percentage of all scheduled treatment procedures were cancelled:

|  | 0% | 1-24% | 25-49% | 50-74% | ≥75% | Don’t know | Not applicable  to my practice |
| --- | --- | --- | --- | --- | --- | --- | --- |
| Cold knife conization | ☐ | ☐ | ☐ | ☐ | ☐ | ☐ | ☐ |
| Other excisional (e.g.,  LEEP) | ☐ | ☐ | ☐ | ☐ | ☐ | ☐ | ☐ |
| Ablative procedures | ☐ | ☐ | ☐ | ☐ | ☐ | ☐ | ☐ |
| Hysterectomy | ☐ | ☐ | ☐ | ☐ | ☐ | ☐ | ☐ |
| Chemotherapy | ☐ | ☐ | ☐ | ☐ | ☐ | ☐ | ☐ |
| Radiation | ☐ | ☐ | ☐ | ☐ | ☐ | ☐ | ☐ |

1. What percentage of all scheduled treatment procedures were postponed:

|  | 0% | 1-24% | 25-49% | 50-74% | ≥75% | Don’t know | Not applicable  to my practice |
| --- | --- | --- | --- | --- | --- | --- | --- |
| Cold knife conization | ☐ | ☐ | ☐ | ☐ | ☐ | ☐ | ☐ |
| Other excisional (e.g.,  LEEP) | ☐ | ☐ | ☐ | ☐ | ☐ | ☐ | ☐ |
| Ablative procedures | ☐ | ☐ | ☐ | ☐ | ☐ | ☐ | ☐ |
| Hysterectomy | ☐ | ☐ | ☐ | ☐ | ☐ | ☐ | ☐ |
| Chemotherapy | ☐ | ☐ | ☐ | ☐ | ☐ | ☐ | ☐ |
| Radiation | ☐ | ☐ | ☐ | ☐ | ☐ | ☐ | ☐ |

1. On average, how long was the deferral period for postponed *(i.e., postponed and/or re-booked)* treatment procedures?

|  | 1 week to  less than 2 weeks | 2 weeks to  less than 4 weeks | 1 month to  less than 2 months | 2 months to less than 4 months | 4 months to less than 6 months | Greater than 6 months | Don’t know | Not applicable to my  practice |
| --- | --- | --- | --- | --- | --- | --- | --- | --- |
| Cold knife  conization | ☐ | ☐ | ☐ | ☐ | ☐ | ☐ | ☐ | ☐ |
| Other excisional  (e.g., LEEP) | ☐ | ☐ | ☐ | ☐ | ☐ | ☐ | ☐ | ☐ |
| Ablative  procedures | ☐ | ☐ | ☐ | ☐ | ☐ | ☐ | ☐ | ☐ |
| Hysterectomy | ☐ | ☐ | ☐ | ☐ | ☐ | ☐ | ☐ | ☐ |
| Chemotherapy | ☐ | ☐ | ☐ | ☐ | ☐ | ☐ | ☐ | ☐ |
| Radiation | ☐ | ☐ | ☐ | ☐ | ☐ | ☐ | ☐ | ☐ |

Telemedicine For the purpose of estimating cumulative effects during the COVID-19 period of interest, please assume it to span from mid-March to mid-August 2020.

1. Has your practice/institution adopted telemedicine to communicate with patients?
   - Yes, with all patients
   - Yes, with low-risk patients only
   - No
   - Don’t know
   - Not applicable to my practice
2. What percentage of patients have you called (audio/video) for distance consultations?
   - 0%
   - 1-24%
   - 25-49%
   - 50-74%
   - ≥75%
   - Don’t know
   - Not applicable to my practice
3. For what percentage of patients have you used telemedicine (audio/video call) during a follow-up appointment related to a cervical cancer screening procedure?
   - 0%
   - 1-24%
   - 25-49%
   - 50-74%
   - ≥75%
   - Don’t know
   - Not applicable to my practice
4. Are virtual consultations compensated for by your jurisdictional public health- insurance system (RAMQ in Quebec, OHIP in Ontario, MSP in BC, etc…)?
   - Yes
   - No
   - Don’t know
   - Not applicable to my practice
5. Which interactions with your patients would be appropriate to convert to telemedicine (audio/video)?
   - Health and medical history reporting
   - Test results reporting
   - Consent forms completion prior to in-person procedure
   - Post-procedure follow-up
   - In-person appointment planning/scheduling
   - Other, *please specify*:

# Over- and under- screening, pre-COVID-19 era

1. In your opinion, in your practice, was there an issue of over-screening for and/or over-diagnosis and/or over-treatment of cervical lesions during the pre-COVID-19 era?

[*Select all that apply*]

- - Yes, over-screening
  - Yes, over-diagnosis
  - Yes, over-treatment
  - No
  - Don’t know

1. Do you think that the current delays/cancellations of screening and management procedures may have had a positive impact by reducing unnecessary screening, diagnosis, and treatment?

[*Select all that apply*]

- - Yes, by reducing over-screening
  - Yes, by reducing over-diagnosis
  - Yes, by reducing over-treatment
  - No
  - Don’t know

1. In your practice, was there an issue of under-screening for and/or under- diagnosis and/or under-treatment of cervical lesions during the pre-COVID- 19 era?

[*Select all that apply*]

- - Yes, under-screening
  - Yes, under-diagnosis
  - Yes, under-treatment
  - No
  - Don’t know

1. Do you think that the current delays/cancellations of screening and management procedures may have had a negative impact by reducing necessary screening, diagnosis, and treatment?

[*Select all that apply*]

- Yes, by reducing screening
- Yes, by reducing diagnosis
- Yes, by reducing treatment
- No
- Don’t know

Resumption of in-person practice Please note that questions 52-61 refer to the impact of the present COVID-19 period (i.e., up to the day you are answering the survey).

1. How long were the services provided in your practice/institution interrupted for, due to the pandemic, before resuming?

- Please provide an answer using units such as weeks and/or months

1. Has your practice/institution caught up with the cancellations/postponements of appointments caused by restrictions introduced at the beginning of the pandemic?
   - Yes
   - No
   - Don’t know
   - Not applicable to my practice
2. Which measures have been implemented by your practice/institution to catch up with these cancellations/postponements?
   - Allow longer workdays and/or working on weekends
   - Increase availability of OR for treatment procedures
   - Convert OR procedures, if possible, to take place in clinics

- Increase availability to labs for processing test samples
- Don’t know
- Not applicable to my practice
- Other, *please specify*:

1. What percentage of cancellations/postponements has your practice/institution currently caught up with?
   - 0%
   - 1-24%
   - 25-49%
   - 50-74%
   - ≥75%
   - Don’t know
   - Not applicable to my practice
2. Have patients been coming in for routine screening procedures at a capacity equivalent to the pre-COVID-19 era?
   - Yes

- No
- Don’t know
- Not applicable to my practice

1. What percentage of patients have been attending routine screening procedures, in comparison to the pre-COVID-19 era?
   - 0%
   - 1-24%
   - 25-49%
   - 50-74%
   - ≥75%
   - Don’t know
   - Not applicable to my practice
2. Have you noticed an increase in the frequency of patients presenting with worsening symptoms (*i.e., unexpected vaginal bleeding, pain during intercourse, etc.*) on routine screening?
   - Yes
   - No
   - Don’t know
   - Not applicable to my practice
3. What percentage of your patients have been diagnosed with more advanced cytological abnormalities and/or lesions confirmed by histology, compared to the pre-COVID-19 era?
   - 0%
   - 1-24%
   - 25-49%
   - 50-74%
   - ≥75%
   - Don’t know
   - Not applicable to my practice
4. Has your practice/institution been screening patients who have been diagnosed with COVID-19?
   - Yes, *briefly describe the process of cervical cancer screening of COVID-19 patients’*
   - No
   - Don’t know
   - Not applicable to my practice
5. Which cervical cancer screening guidelines has your practice/institution been following (please be specific as to the source, e.g., professional society, cancer organization, provincial directive, local hospital instructions)?

Finally, please fill the following information about yourself:

What is your sex?

- Male
- Female
- Other
- Prefer not to say

What is your age: years

- Leave blank if you prefer not to give us this information

Do you have any additional comments and/or concerns relating to the survey?

END OF SURVEY

Thank you for taking the time to complete this survey

Survey development McGill team:

Rami Ali, Eliya Farah, Mariam El-Zein, Eduardo Franco

Canadian Experts in the field of cervical cancer and prevention management: Aisha Lofters, Barbara Bodmer, Celine Bouchard, CM McLachlin, Fady Williamson Mansour, Gina Ogilvie, James Bentley, Jennifer Blake, Jessica Ann Papillon Smith, Joan Murphy, Kathleen Decker, Lorraine (Laurie) Elit, Lucy Gilbert, Maire A. Duggan, Marette Lee, Marie-Helene Mayrand, Paul Bessette, Rachel Kupets, Shannon Salvador, Suzie Lau, Walter Henri Gotlieb
