# Supplementary material for: Pan-Canadian survey on the impact of the COVID-19 pandemic on cervical cancer screening and management: cross-sectional survey of healthcare professionals
Source: eLife. 2023 Jun 28;12:e83764. doi: 10.7554/eLife.83764 (PMC10368419; doi:10.7554/eLife.83764)
Supplement: Supplementary file 2. — Includes frequency distribution of Q11-13 (2a), Q14-16 (2b), Q17-19 (2c), Q20-21 (2d), Q22-23 (2e), Q24-29 (2f), Q30-35 (2g), Q36-37 (2h), and Q38-Q42 (2i). [file elife-83764-supp2.docx]

**Supplementary File 2**

**Theme 1: Screening practice**

**Supplementary File 2a.** Pandemic-related updates to practice/institutional/jurisdictional procedures (n = 510)

| **Question number & content** | **Categories** | **n (%)** |
| --- | --- | --- |
| Q11 Allowance of **in-person consultations** during peak pandemic | Yes | 378 (74.1) |
|  | No | 99 (19.4) |
|  | *Don’t know* | *13 (2.6)* |
|  | *Not applicable to my practice* | *20 (3.9)* |
| Q12 Informed about **latest recommendations/guidelines** for cervical cancer screening during peak pandemic | Yes | 274 (53.7) |
|  | No | 164 (32.2) |
|  | *Don’t know* | *62 (12.2)* |
|  | *Not applicable to my practice* | *10 (2.0)* |
| Q13 **Frequency of updates** to recommendations/guidelines that modify scheduling/performing cervical cancer screening | Every week | 18 (3.5) |
|  | Every 2 weeks | 48 (9.4) |
|  | Every month | 101 (19.8) |
|  | Every 2 months | 58 (11.4) |
|  | >2 months | 118 (23.1) |
|  | Never | 92 (18.0) |
|  | *Don’t know* | *65 (12.8)* |
|  | *Not applicable to my practice* | *10 (2.0)* |

**Supplementary File 2b.** Screening test practices (n = 469)

| **Question number & content** | | **Categories** | **n (%)** |
| --- | --- | --- | --- |
| Q14 **Type of test** used for primary cervical cancer screening | **Pap** test | Yes | 357 (76.1) |
|  |  | No | 61 (13.0) |
|  |  | *Don’t know* | *37 (7.9)* |
|  |  | *Not applicable to my practice* | *14 (3.0)* |
|  | **HPV** test | Yes | 154 (32.8) |
|  |  | No | 230 (49.0) |
|  |  | *Don’t know* | *36 (7.7)* |
|  |  | *Not applicable to my practice* | *49 (10.5)* |
|  | **HPV/Pap** co-test | Yes | 119 (25.4) |
|  |  | No | 262 (55.9) |
|  |  | *Don’t know* | 43 (9.2) |
|  |  | *Not applicable to my practice* | 45 (9.6) |
| **Q15** **Change in number of screening tests** compared to pre-COVID-19 | **Pap** test | Decrease ≥75% | 74 (15.8) |
|  |  | Decrease 26-74% | 117 (25.0) |
|  |  | Decrease ≤25% | 95 (20.3) |
|  |  | Unaffected | 82 (17.5) |
|  |  | Increase ≤25% | 24 (5.1) |
|  |  | Increase 26-74% | 18 (3.8) |
|  |  | Increase ≥75% | 9 (1.9) |
|  |  | *Don’t know* | *37 (7.9)* |
|  |  | *Not applicable to my practice* | *13 (2.8)* |
|  | **HPV** test | Decrease ≥75% | 23 (4.9) |
|  |  | Decrease 26-74% | 31 (6.6) |
|  |  | Decrease ≤25% | 33 (7.0) |
|  |  | Unaffected | 91 (19.4) |
|  |  | Increase ≤25% | 34 (7.3) |
|  |  | Increase 26-74% | 30 (6.4) |
|  |  | Increase ≥75% | 11 (2.4) |
|  |  | *Don’t know* | *43 (9.2)* |
|  |  | *Not applicable to my practice* | *173 (37.0)* |
|  | **HPV/Pap** co-test | Decrease ≥75% | 18 (3.8) |
|  |  | Decrease 26-74% | 36 (7.7) |
|  |  | Decrease ≤25% | 35 (7.5) |
|  |  | Unaffected | 70 (14.9) |
|  |  | Increase ≤25% | 36 (7.7) |
|  |  | Increase 26-74% | 22 (4.7) |
|  |  | Increase ≥75% | 12 (2.6) |
|  |  | *Don’t know* | *39 (8.3)* |
|  |  | *Not applicable to my practice* | *201 (42.9)* |
| **Q16 Delays** in scheduling of **tests** | **Pap** test | Yes | 267 (56.9) |
|  |  | No | 132 (28.1) |
|  |  | *Don’t know* | *50 (10.7)* |
|  |  | *Not applicable to my practice* | *20 (4.3)* |
|  | **HPV** test | Yes | 106 (22.6) |
|  |  | No | 116 (24.7) |
|  |  | *Don’t know* | 57 (12.2) |
|  |  | *Not applicable to my practice* | 190 (40.5) |
|  | **HPV/Pap** co-test | Yes | 102 (21.8) |
|  |  | No | 93 (19.8) |
|  |  | *Don’t know* | *58 (12.4)* |
|  |  | *Not applicable to my practice* | *216 (46.1)* |

HPV: human papillomavirus

**Supplementary File 2c.** Cancellation and postponement of scheduled screening tests by type of test

| **Question number & content** (number of responses) | | **Categories** | **n (%)** |
| --- | --- | --- | --- |
| Q17 Percentage of **scheduled screening tests** that have been **cancelled** (n = 469) | **Pap** test | 0% | 76 (16.2) |
|  |  | 1-24% | 145 (31.0) |
|  |  | 25-49% | 80 (17.1) |
|  |  | 50-74% | 45 (9.6) |
|  |  | ≥75% | 49 (10.5) |
|  |  | *Don’t know* | *54 (11.5)* |
|  |  | *Not applicable to my practice* | *20 (4.3)* |
|  | **HPV** test | 0% | 65 (13.9) |
|  |  | 1-24% | 41 (8.7) |
|  |  | 25-49% | 52 (11.1) |
|  |  | 50-74% | 33 (7.0) |
|  |  | ≥75% | 37 (7.9) |
|  |  | *Don’t know* | *56 (11.9)* |
|  |  | *Not applicable to my practice* | *185 (39.5)* |
|  | **HPV/Pap** co-test | 0% | 49 (10.5) |
|  |  | 1-24% | 43 (9.2) |
|  |  | 25-49% | 38 (8.1) |
|  |  | 50-74% | 39 (8.3) |
|  |  | ≥75% | 42 (9.0) |
|  |  | *Don’t know* | *45 (9.6)* |
|  |  | *Not applicable to my practice* | *213 (45.4)* |
| Q18 Percentage of **scheduled screening tests** that have been **postponed** (n = 468) | **Pap** test | 0% | 29 (6.2) |
|  |  | 1-24% | 119 (25.4) |
|  |  | 25-49% | 100 (21.4) |
|  |  | 50-74% | 75 (16.0) |
|  |  | ≥75% | 78 (16.7) |
|  |  | *Don’t know* | *48 (10.3)* |
|  |  | *Not applicable to my practice* | *19 (4.1)* |
|  | **HPV** test | 0% | 49 (10.5) |
|  |  | 1-24% | 49 (10.5) |
|  |  | 25-49% | 55 (11.8) |
|  |  | 50-74% | 36 (7.7) |
|  |  | ≥75% | 45 (9.6) |
|  |  | *Don’t know* | *47 (10.0)* |
|  |  | *Not applicable to my practice* | *187 (40.0)* |
|  | **HPV/Pap** co-test | 0% | 44 (9.4) |
|  |  | 1-24% | 45 (9.6) |
|  |  | 25-49% | 49 (10.5) |
|  |  | 50-74% | 43 (9.2) |
|  |  | ≥75% | 32 (6.8) |
|  |  | *Don’t know* | *45 (9.6)* |
|  |  | *Not applicable to my practice* | *210 (44.9)* |
| **Q19 Deferral** period of postponed **testing appointments** (n = 467) | **Pap** test | 1 week to <2 weeks | 15 (3.2) |
|  |  | 2 weeks to <4 weeks | 43 (9.2) |
|  |  | 1 month to <2 months | 78 (16.7) |
|  |  | 2 months to <4 months | 128 (27.4) |
|  |  | 4 months to <6 months | 87 (18.7) |
|  |  | >6 months | 47 (10.1) |
|  |  | *Don’t know* | 36 (7.7) |
|  |  | *Not applicable to my practice* | 33 (7.1) |
|  | **HPV** test | 1 week to <2 weeks | 22 (4.7) |
|  |  | 2 weeks to <4 weeks | 31 (6.6) |
|  |  | 1 month to <2 months | 46 (9.9) |
|  |  | 2 months to <4 months | 48 (10.3) |
|  |  | 4 months to <6 months | 35 (7.5) |
|  |  | >6 months | 31 (6.6) |
|  |  | *Don’t know* | 42 (9.0) |
|  |  | *Not applicable to my practice* | 212 (45.4) |
|  | **HPV/Pap** co-test | 1 week to <2 weeks | 16 (3.4) |
|  |  | 2 weeks to <4 weeks | 27 (5.8) |
|  |  | 1 month to <2 months | 30 (6.4) |
|  |  | 2 months to <4 months | 46 (9.9) |
|  |  | 4 months to <6 months | 47 (10.1) |
|  |  | >6 months | 31 (6.6) |
|  |  | *Don’t know* | 36 (7.7) |
|  |  | *Not applicable to my practice* | 234 (50.1) |

HPV: human papillomavirus

**Supplementary File 2d.** Pandemic-related delays in forwarding screening test samples to the lab (n = 467)

| **Question number & content** | | **Categories** | **n (%)** |
| --- | --- | --- | --- |
| **Q20 Delay** in **forwarding tests to labs** for processing | **Pap** test | Yes | 70 (15.0) |
|  |  | No | 323 (69.2) |
|  |  | *Don’t know* | *57 (12.2)* |
|  |  | *Not applicable to my practice* | *17 (3.6)* |
|  | **HPV** test | Yes | 73 (15.6) |
|  |  | No | 164 (35.1) |
|  |  | *Don’t know* | *42 (9.0)* |
|  |  | *Not applicable to my practice* | *188 (40.3)* |
|  | **HPV/Pap** co-test | Yes | 65 (13.9) |
|  |  | No | 126 (27.0) |
|  |  | *Don’t know* | *58 (12.4)* |
|  |  | *Not applicable to my practice* | *218 (46.7)* |
| **Q21 Delay** period in **forwarding test** samples to lab | **Pap** test | 1 week to <2 weeks | 73 (15.6) |
|  |  | 2 weeks to <4 weeks | 32 (6.9) |
|  |  | 1 month to <2 months | 45 (9.6) |
|  |  | 2 months to <4 months | 39 (8.4) |
|  |  | 4 months to <6 months | 24 (5.1) |
|  |  | >6 months | 6 (1.3) |
|  |  | *Don’t know* | *63 (13.5)* |
|  |  | *Not applicable to my practice* | *185 (39.6)* |
|  | **HPV** test | 1 week to <2 weeks | 35 (7.5) |
|  |  | 2 weeks to <4 weeks | 29 (6.2) |
|  |  | 1 month to <2 months | 32 (6.9) |
|  |  | 2 months to <4 months | 28 (6.0) |
|  |  | 4 months to <6 months | 34 (7.3) |
|  |  | >6 months | 22 (4.7) |
|  |  | *Don’t know* | *40 (8.6)* |
|  |  | *Not applicable to my practice* | *247 (52.9)* |
|  | **HPV/Pap** co-test | 1 week to <2 weeks | 32 (6.9) |
|  |  | 2 weeks to <4 weeks | 25 (5.4) |
|  |  | 1 month to <2 months | 24 (5.1) |
|  |  | 2 months to <4 months | 37 (7.9) |
|  |  | 4 months to <6 months | 27 (5.8) |
|  |  | >6 months | 18 (3.9) |
|  |  | *Don’t know* | *43 (9.2)* |
|  |  | *Not applicable to my practice* | *261 (55.9)* |

HPV: human papillomavirus

**Supplementary File 2e.** Professional opinions on HPV self-sampling (n = 455)

| **Question number & content** | **Categories** | **n (%)** |
| --- | --- | --- |
| Q22 **COVID-19** to encourage/facilitate/accelerate **implementation of HPV self-sampling** in cervical cancer screening programs | Yes | 150 (33.0) |
|  | No | 143 (31.4) |
|  | Maybe | 162 (35.6) |
| Q23 **In favor** of implementing **HPV self-sampling** as alternative screening method in practice | Yes | 228 (50.1) |
|  | No | 123 (27.0) |
|  | Maybe | 104 (22.9) |

HPV: human papillomavirus

**Supplementary File 2f.** Changes in colposcopy appointments

| **Question number & content** (number of responses) | | **Categories** | **n (%)** |
| --- | --- | --- | --- |
| Q24 **Changes in number of colposcopy-biopsy** procedures compared to pre-COVID-19  (n = 452) | | Decrease ≥75% | 21 (4.7) |
|  |  | Decrease 26-74% | 61 (13.5) |
|  |  | Decrease ≤25% | 87 (19.3) |
|  |  | Unaffected | 40 (8.9) |
|  |  | Increase ≤25% | 24 (5.3) |
|  |  | Increase 26-74% | 10 (2.2) |
|  |  | Increase ≥75% | 6 (1.3) |
|  |  | *Don’t know* | *34 (7.5)* |
|  |  | *Not applicable to my practice* | *169 (37.4)* |
| Q25 **Cancellations** of **colposcopy** appointments  (n = 452) | | Yes | 114 (25.2) |
|  |  | No | 95 (21.0) |
|  |  | *Don’t know* | *72 (15.9)* |
|  |  | *Not applicable to my practice* | *171 (37.8)* |
| Q26 Percentage of **cancelled colposcopy** appointments  (n = 114) | Cancelled by **physician or provider’s institution** | 0% | 5 (4.4) |
|  |  | 1-24% | 45 (39.5) |
|  |  | 25-49% | 30 (26.3) |
|  |  | 50-74% | 16 (14.0) |
|  |  | ≥75% | 11 (9.7) |
|  |  | *Don’t know* | *7 (6.1)* |
|  | Cancelled by **patient** | 0% | 6 (5.3) |
|  |  | 1-24% | 41 (36.0) |
|  |  | 25-49% | 23 (20.2) |
|  |  | 50-74% | 21 (18.4) |
|  |  | ≥75% | 14 (12.3) |
|  |  | *Don’t know* | *9 (7.9)* |
| Q27 **Postponements** in scheduling of **colposcopy appointments**  (n = 452) | | Yes | 167 (37.0) |
|  |  | No | 68 (15.0) |
|  |  | *Don’t know* | *44 (9.7)* |
|  |  | *Not applicable to my practice* | *173 (38.3)* |
| Q28 Percentage of **postponed colposcopy** appointments  (n = 167) | Postponed by **physician or provider’s institution** | 0% | 2 (1.2) |
|  |  | 1-24% | 47 (28.1) |
|  |  | 25-49% | 40 (24.0) |
|  |  | 50-74% | 43 (25.8) |
|  |  | ≥75% | 25 (15.0) |
|  |  | *Don’t know* | *10 (6.0)* |
|  | Postponed by **patient** | 0% | 7 (4.2) |
|  |  | 1-24% | 62 (37.1) |
|  |  | 25-49% | 44 (26.4) |
|  |  | 50-74% | 23 (13.8) |
|  |  | ≥75% | 14 (8.4) |
|  |  | *Don’t know* | *17 (10.2)* |
| **Q29 Length of deferral** period of postponed **colposcopy appointments**  (n = 167) | | 1 week to <2 weeks | 6 (3.6) |
|  |  | 2 weeks to <4 weeks | 18 (10.8) |
|  |  | 1 month to <2 months | 44 (26.4) |
|  |  | 2 months to <4 months | 59 (35.3) |
|  |  | 4 months to <6 months | 25 (15.0) |
|  |  | >6 months | 9 (5.4) |
|  |  | *Don’t know* | *6 (3.6)* |

**Supplementary File 2g.** Changes in follow-up appointments

| **Question number & content** (number of responses) | | | **Categories** | **n (%)** |
| --- | --- | --- | --- | --- |
| Q30 **Delay in follow-up** of patients following test results  (n = 446) | | Abnormal cytology | Yes | 121 (27.1) |
|  |  |  | No | 251 (56.3) |
|  |  |  | *Don’t know* | 52 (11.7) |
|  |  |  | *Not applicable to my practice* | 22 (4.9) |
|  |  | High-grade lesions | Yes | 97 (21.8) |
|  |  |  | No | 257 (57.6) |
|  |  |  | *Don’t know* | 57 (12.8) |
|  |  |  | *Not applicable to my practice* | 35 (7.9) |
|  |  | Positive HPV test | Yes | 75 (16.8) |
|  |  |  | No | 191 (42.8) |
|  |  |  | *Don’t know* | 58 (13.0) |
|  |  |  | *Not applicable to my practice* | 122 (27.4) |
|  |  | Positive HPV self-sampling | Yes | 55 (12.3) |
|  |  |  | No | 96 (21.5) |
|  |  |  | *Don’t know* | 52 (11.7) |
|  |  |  | *Not applicable to my practice* | 243 (54.5) |
| Q31 **Cancellations** of **follow-up** appointments  (n = 445) | | | Yes | 148 (33.3) |
|  |  |  | No | 170 (38.2) |
|  |  |  | *Don’t know* | 97 (21.8) |
|  |  |  | *Not applicable to my practice* | 30 (6.7) |
| Q32 Percentage of **cancelled follow-up** appointments  (n = 148) | | Cancelled by **physician or provider’s institution** | 0% | 25 (16.9) |
|  |  |  | 1-24% | 58 (39.2) |
|  |  |  | 25-49% | 29 (19.6) |
|  |  |  | 50-74% | 19 (12.8) |
|  |  |  | ≥75% | 9 (6.1) |
|  |  |  | *Don’t know* | 8 (5.4) |
|  |  | Cancelled by **patient** | 0% | 3 (2.0) |
|  |  |  | 1-24% | 61 (41.2) |
|  |  |  | 25-49% | 35 (23.7) |
|  |  |  | 50-74% | 28 (18.9) |
|  |  |  | ≥75% | 15 (10.1) |
|  |  |  | *Don’t know* | 6 (4.1) |
|  |  | **Converted to telemedicine** | 0% | 24 (16.2) |
|  |  |  | 1-24% | 44 (29.7) |
|  |  |  | 25-49% | 29 (19.6) |
|  |  |  | 50-74% | 26 (17.6) |
|  |  |  | ≥75% | 14 (9.5) |
|  |  |  | *Don’t know* | 11 (7.4) |
| **Q33 Postponements** in scheduling of **follow-up appointments**  (n = 445) | | | Yes | 238 (53.5) |
|  |  |  | No | 123 (27.6) |
|  |  |  | *Don’t know* | 55 (12.4) |
|  |  |  | *Not applicable to my practice* | 29 (6.5) |
| Q34 Percentage of **postponed follow-up** appointments (n=238) | Postponed by **physician or provider’s institution** | | 0% | 21 (8.8) |
|  |  |  | 1-24% | 84 (35.3) |
|  |  |  | 25-49% | 49 (20.6) |
|  |  |  | 50-74% | 40 (16.8) |
|  |  |  | ≥75% | 28 (11.8) |
|  |  |  | *Don’t know* | *16 (6.7)* |
|  | Postponed by **patient** | | 0% | 6 (2.5) |
|  |  |  | 1-24% | 98 (41.2) |
|  |  |  | 25-49% | 59 (24.8) |
|  |  |  | 50-74% | 36 (15.1) |
|  |  |  | ≥75% | 19 (8.0) |
|  |  |  | *Don’t know* | *20 (8.4)* |
|  | **Converted to telemedicine** | | 0% | 48 (20.2) |
|  |  |  | 1-24% | 51 (21.4) |
|  |  |  | 25-49% | 41 (17.2) |
|  |  |  | 50-74% | 42 (17.6) |
|  |  |  | ≥75% | 29 (12.2) |
|  |  |  | *Don’t know* | *27 (11.3)* |
| **Q35 Length of deferral** period of postponed **follow-up appointments** (n=238) | | | 1 week to <2 weeks | 11 (4.6) |
|  |  |  | 2 weeks to <4 weeks | 39 (16.4) |
|  |  |  | 1 month to <2 months | 67 (28.2) |
|  |  |  | 2 months to <4 months | 69 (29.0) |
|  |  |  | 4 months to <6 months | 35 (14.7) |
|  |  |  | >6 months | 9 (3.8) |
|  |  |  | *Don’t know* | *8 (3.4)* |

HPV: human papillomavirus

**Supplementary File 2h.** Pandemic-related delays in receiving test results from lab (n = 445)

| **Question number & content** | | **Categories** | **n (%)** |
| --- | --- | --- | --- |
| Q36 **Delay in receiving test results** from lab | **Pap** test | Yes | 94 (21.1) |
|  |  | No | 273 (61.4) |
|  |  | *Don’t know* | 65 (14.6) |
|  |  | *Not applicable to my practice* | 13 (2.9) |
|  | **HPV** test | Yes | 86 (19.3) |
|  |  | No | 130 (29.2) |
|  |  | *Don’t know* | 63 (14.2) |
|  |  | *Not applicable to my practice* | 166 (37.3) |
|  | **HPV/Pap** co-test | Yes | 54 (12.1) |
|  |  | No | 116 (26.1) |
|  |  | *Don’t know* | 76 (17.1) |
|  |  | *Not applicable to my practice* | 199 (44.7) |
| **Q37 Delay** period in **receiving test** samples from lab | **Pap** test | 1 week to <2 weeks | 62 (13.9) |
|  |  | 2 weeks to <4 weeks | 49 (11.0) |
|  |  | 1 month to <2 months | 58 (13.0) |
|  |  | 2 months to <4 months | 45 (10.1) |
|  |  | 4 months to <6 months | 28 (6.3) |
|  |  | >6 months | 15 (3.4) |
|  |  | *Don’t know* | 56 (12.6) |
|  |  | *Not applicable to my practice* | 132 (29.7) |
|  | **HPV** test | 1 week to <2 weeks | 30 (6.8) |
|  |  | 2 weeks to <4 weeks | 31 (7.0) |
|  |  | 1 month to <2 months | 37 (8.3) |
|  |  | 2 months to <4 months | 35 (7.9) |
|  |  | 4 months to <6 months | 35 (7.9) |
|  |  | >6 months | 17 (3.8) |
|  |  | *Don’t know* | 42 (9.4) |
|  |  | *Not applicable to my practice* | 218 (49.0) |
|  | **HPV/Pap** co-test | 1 week to <2 weeks | 29 (6.5) |
|  |  | 2 weeks to <4 weeks | 23 (5.2) |
|  |  | 1 month to <2 months | 28 (6.3) |
|  |  | 2 months to <4 months | 32 (7.2) |
|  |  | 4 months to <6 months | 31 (7.0) |
|  |  | >6 months | 19 (4.3) |
|  |  | *Don’t know* | 51 (11.5) |
|  |  | *Not applicable to my practice* | 232 (52.1) |

HPV: human papillomavirus

**Theme 2: Treatment of pre-cancerous lesions and cancer**

**Supplementary File 2i.** Changes observed in number of treatment procedures, by treatment type (n = 431)

| **Variable** | | **Categories** | **n (%)** |
| --- | --- | --- | --- |
| Q38 Changes in **number of treatment procedures** | Cold knife conization | Decrease ≥75% | 14 (3.3) |
|  |  | Decrease 26-74% | 25 (5.8) |
|  |  | Decrease ≤25% | 29 (6.7) |
|  |  | Unaffected | 54 (12.5) |
|  |  | Increase ≤25% | 28 (6.5) |
|  |  | Increase 26-74% | 27 (6.3) |
|  |  | Increase ≥75% | 13 (3.0) |
|  |  | *Don’t know* | *41 (9.5)* |
|  |  | *Not applicable to my practice* | *200 (46.4)* |
|  | Other excisional | Decrease ≥75% | 14 (3.3) |
|  |  | Decrease 26-74% | 32 (7.4) |
|  |  | Decrease ≤25% | 40 (9.3) |
|  |  | Unaffected | 55 (12.8) |
|  |  | Increase ≤25% | 32 (7.4) |
|  |  | Increase 26-74% | 23 (5.3) |
|  |  | Increase ≥75% | 12 (2.8) |
|  |  | *Don’t know* | *34 (7.9)* |
|  |  | *Not applicable to my practice* | *189 (43.9)* |
|  | Ablative procedures | Decrease ≥75% | 15 (3.5) |
|  |  | Decrease 26-74% | 24 (5.6) |
|  |  | Decrease ≤25% | 29 (6.7) |
|  |  | Unaffected | 47 (10.9) |
|  |  | Increase ≤25% | 31 (7.2) |
|  |  | Increase 26-74% | 24 (5.6) |
|  |  | Increase ≥75% | 15 (3.5) |
|  |  | *Don’t know* | *36 (8.4)* |
|  |  | *Not applicable to my practice* | *210 (48.7)* |
|  | Hysterectomy | Decrease ≥75% | 20 (4.6) |
|  |  | Decrease 26-74% | 36 (8.4) |
|  |  | Decrease ≤25% | 47 (10.9) |
|  |  | Unaffected | 40 (9.3) |
|  |  | Increase ≤25% | 26 (6.0) |
|  |  | Increase 26-74% | 19 (4.4) |
|  |  | Increase ≥75% | 13 (3.0) |
|  |  | *Don’t know* | *37 (8.6)* |
|  |  | *Not applicable to my practice* | *193 (44.8)* |
|  | Chemotherapy | Decrease ≥75% | 6 (1.4) |
|  |  | Decrease 26-74% | 12 (2.8) |
|  |  | Decrease ≤25% | 29 (6.7) |
|  |  | Unaffected | 47 (10.9) |
|  |  | Increase ≤25% | 30 (7.0) |
|  |  | Increase 26-74% | 19 (4.4) |
|  |  | Increase ≥75% | 16 (3.7) |
|  |  | *Don’t know* | *40 (9.3)* |
|  |  | *Not applicable to my practice* | *232 (53.8)* |
|  | Radiation | Decrease ≥75% | 7 (1.6) |
|  |  | Decrease 26-74% | 13 (3.0) |
|  |  | Decrease ≤25% | 39 (9.1) |
|  |  | Unaffected | 34 (7.9) |
|  |  | Increase ≤25% | 27 (6.3) |
|  |  | Increase 26-74% | 21 (4.9) |
|  |  | Increase ≥75% | 12 (2.8) |
|  |  | *Don’t know* | *42 (9.7)* |
|  |  | *Not applicable to my practice* | *236 (54.8)* |
| Q39 **Cancellations / postponements of treatment procedures** | Cold knife conization | Yes | 58 (13.5) |
|  |  | No | 99 (23.0) |
|  |  | *Don’t know* | *63 (14.6)* |
|  |  | *Not applicable to my practice* | *211 (49.0)* |
|  | Other excisional | Yes | 102 (23.7) |
|  |  | No | 78 (18.1) |
|  |  | *Don’t know* | *62 (14.4)* |
|  |  | *Not applicable to my practice* | *188 (43.9)* |
|  | Ablative procedures | Yes | 84 (19.5) |
|  |  | No | 81 (18.8) |
|  |  | *Don’t know* | *54 (12.5)* |
|  |  | *Not applicable to my practice* | *212 (49.2)* |
|  | Hysterectomy | Yes | 91 (21.1) |
|  |  | No | 79 (18.3) |
|  |  | *Don’t know* | *61 (14.2)* |
|  |  | *Not applicable to my practice* | *200 (46.4)* |
|  | Chemotherapy | Yes | 45 (10.4) |
|  |  | No | 82 (19.0) |
|  |  | *Don’t know* | *69 (16.0)* |
|  |  | *Not applicable to my practice* | *235 (54.5)* |
|  | Radiation | Yes | 50 (11.6) |
|  |  | No | 72 (16.7) |
|  |  | *Don’t know* | *72 (16.7)* |
|  |  | *Not applicable to my practice* | *237 (55.0)* |
| Q40 Percentage of **scheduled treatment procedures** that were **cancelled** | Cold knife conization | 0% | 37 (8.6) |
|  |  | 1-24% | 34 (7.9) |
|  |  | 25-49% | 47 (10.9) |
|  |  | 50-74% | 29 (6.7) |
|  |  | ≥75% | 29 (6.7) |
|  |  | *Don’t know* | *52 (12.1)* |
|  |  | *Not applicable to my practice* | *203 (47.3)* |
|  | Other excisional | 0% | 44 (10.2) |
|  |  | 1-24% | 43 (10.0) |
|  |  | 25-49% | 47 (10.9) |
|  |  | 50-74% | 40 (9.3) |
|  |  | ≥75% | 22 (5.1) |
|  |  | *Don’t know* | *50 (11.6)* |
|  |  | *Not applicable to my practice* | *185 (42.9)* |
|  | Ablative procedures | 0% | 35 (8.1) |
|  |  | 1-24% | 22 (5.1) |
|  |  | 25-49% | 49 (11.4) |
|  |  | 50-74% | 31 (7.2) |
|  |  | ≥75% | 30 (7.0) |
|  |  | *Don’t know* | *59 (13.7)* |
|  |  | *Not applicable to my practice* | *205 (47.6)* |
|  | Hysterectomy | 0% | 40 (9.3) |
|  |  | 1-24% | 36 (8.4) |
|  |  | 25-49% | 40 (9.3) |
|  |  | 50-74% | 35 (8.1) |
|  |  | ≥75% | 31 (7.2) |
|  |  | *Don’t know* | *54 (12.5)* |
|  |  | *Not applicable to my practice* | *195 (45.2)* |
|  | Chemotherapy | 0% | 20 (4.7) |
|  |  | 1-24% | 36 (8.4) |
|  |  | 25-49% | 36 (8.4) |
|  |  | 50-74% | 34 (7.9) |
|  |  | ≥75% | 20 (4.6) |
|  |  | *Don’t know* | *57 (13.2)* |
|  |  | *Not applicable to my practice* | *228 (52.9)* |
|  | Radiation | 0% | 21 (4.9) |
|  |  | 1-24% | 33 (7.7) |
|  |  | 25-49% | 36 (8.4) |
|  |  | 50-74% | 32 (7.4) |
|  |  | ≥75% | 26 (6.0) |
|  |  | *Don’t know* | *53 (12.3)* |
|  |  | *Not applicable to my practice* | *230 (53.4)* |
| Q41 Percentage of **scheduled treatment procedures** that were **postponed** | Cold knife conization | 0% | 26 (6.0) |
|  |  | 1-24% | 46 (10.7) |
|  |  | 25-49% | 46 (10.7) |
|  |  | 50-74% | 37 (8.6) |
|  |  | ≥75% | 20 (4.7) |
|  |  | *Don’t know* | *52 (12.1)* |
|  |  | *Not applicable to my practice* | *204 (47.3)* |
|  | Other excisional | 0% | 24 (5.6) |
|  |  | 1-24% | 57 (13.2) |
|  |  | 25-49% | 48 (11.1) |
|  |  | 50-74% | 37 (8.6) |
|  |  | ≥75% | 27 (6.3) |
|  |  | *Don’t know* | *53 (12.3)* |
|  |  | *Not applicable to my practice* | *185 (42.9)* |
|  | Ablative procedures | 0% | 20 (4.6) |
|  |  | 1-24% | 42 (9.7) |
|  |  | 25-49% | 37 (8.6) |
|  |  | 50-74% | 37 (8.6) |
|  |  | ≥75% | 35 (8.1) |
|  |  | *Don’t know* | *54 (12.5)* |
|  |  | *Not applicable to my practice* | *206 (47.8)* |
|  | Hysterectomy | 0% | 21 (4.9) |
|  |  | 1-24% | 44 (10.2) |
|  |  | 25-49% | 45 (10.4) |
|  |  | 50-74% | 41 (9.5) |
|  |  | ≥75% | 27 (6.3) |
|  |  | *Don’t know* | *59 (13.7)* |
|  |  | *Not applicable to my practice* | *194 (45.0)* |
|  | Chemotherapy | 0% | 20 (4.6) |
|  |  | 1-24% | 39 (9.1) |
|  |  | 25-49% | 40 (9.3) |
|  |  | 50-74% | 25 (5.8) |
|  |  | ≥75% | 26 (6.0) |
|  |  | *Don’t know* | *55 (12.8)* |
|  |  | *Not applicable to my practice* | *226 (52.4)* |
|  | Radiation | 0% | 19 (4.4) |
|  |  | 1-24% | 32 (7.4) |
|  |  | 25-49% | 33 (7.7) |
|  |  | 50-74% | 37 (8.6) |
|  |  | ≥75% | 21 (4.9) |
|  |  | *Don’t know* | *62 (14.4)* |
|  |  | *Not applicable to my practice* | *227 (52.7)* |
| Q42 Length of **deferral period** for **postponed** **treatment procedures** | Cold knife conization | 1 week to <2 weeks | 13 (3.0) |
|  |  | 2 weeks to <4 weeks | 31 (7.2) |
|  |  | 1 month to <2 months | 32 (7.4) |
|  |  | 2 months to <4 months | 42 (9.7) |
|  |  | 4 months to <6 months | 33 (7.7) |
|  |  | >6 months | 13 (3.0) |
|  |  | *Don’t know* | *48 (11.1)* |
|  |  | *Not applicable to my practice* | *219 (50.8)* |
|  | Other excisional | 1 week to <2 weeks | 19 (4.4) |
|  |  | 2 weeks to <4 weeks | 32 (7.4) |
|  |  | 1 month to <2 months | 52 (12.1) |
|  |  | 2 months to <4 months | 33 (7.7) |
|  |  | 4 months to <6 months | 33 (7.7) |
|  |  | >6 months | 16 (3.7) |
|  |  | *Don’t know* | *48 (11.1)* |
|  |  | *Not applicable to my practice* | *198 (45.9)* |
|  | Ablative procedures | 1 week to <2 weeks | 17 (3.9) |
|  |  | 2 weeks to <4 weeks | 18 (4.2) |
|  |  | 1 month to <2 months | 40 (9.3) |
|  |  | 2 months to <4 months | 42 (9.7) |
|  |  | 4 months to <6 months | 30 (7.0) |
|  |  | >6 months | 20 (4.6) |
|  |  | *Don’t know* | *50 (11.6)* |
|  |  | *Not applicable to my practice* | *214 (49.7)* |
|  | Hysterectomy | 1 week to <2 weeks | 21 (4.9) |
|  |  | 2 weeks to <4 weeks | 23 (5.3) |
|  |  | 1 month to <2 months | 31 (7.2) |
|  |  | 2 months to <4 months | 46 (10.7) |
|  |  | 4 months to <6 months | 30 (7.0) |
|  |  | >6 months | 20 (4.6) |
|  |  | *Don’t know* | *55 (12.8)* |
|  |  | *Not applicable to my practice* | *205 (47.6)* |
|  | Chemotherapy | 1 week to <2 weeks | 19 (4.4) |
|  |  | 2 weeks to <4 weeks | 28 (6.5) |
|  |  | 1 month to <2 months | 32 (7.4) |
|  |  | 2 months to <4 months | 27 (6.3) |
|  |  | 4 months to <6 months | 28 (6.5) |
|  |  | >6 months | 22 (5.1) |
|  |  | *Don’t know* | *49 (11.4)* |
|  |  | *Not applicable to my practice* | *226 (52.4)* |
|  | Radiation | 1 week to <2 weeks | 17 (3.9) |
|  |  | 2 weeks to <4 weeks | 28 (6.5) |
|  |  | 1 month to <2 months | 29 (6.7) |
|  |  | 2 months to <4 months | 36 (8.4) |
|  |  | 4 months to <6 months | 25 (5.8) |
|  |  | >6 months | 11 (2.6) |
|  |  | *Don’t know* | *55 (12.8)* |
|  |  | *Not applicable to my practice* | *230 (53.4)* |
